# Congenital hyperinsulinism associated with Hirschsprung’s disease—a report of an extremely rare case

**DOI:** 10.1186/s40792-020-0778-3

**Published:** 2020-01-08

**Authors:** Takeshi Shono, Kumiko Shono, Yoshiko Hashimoto, Shohei Taguchi, Masanori Masuda, Kastumi Muramori, Tomoaki Taguchi

**Affiliations:** 1 Department of Pediatric Surgery, National Hospital Organization, Kokura Medical Center, Harugaoka 10-1, Kokuraminami-Ku, Kitakyushu, 803-8533 Japan; 2 Department of Pediatric Surgery, Kitakyushu Medical Center, Kitakyushu, Japan; 3 Department of Pathology, Saga-ken Medical Center Koseikan, Saga, Japan; 4 Department of Pediatric Surgery, Saga-ken Medical Center Koseikan, Saga, Japan; 5 Department of Pediatric Surgery, Kyushu University, Fukuoka, Japan

**Keywords:** Congenital hyperinsulinism, Nesidioblastosis, Hirschsprung’s disease, Diazoxide, Octreotide

## Abstract

**Background:**

Congenital hyperinsulinism (CH) is a rare disease, characterized by severe hypoglycemia induced by inappropriate insulin secretion from pancreatic beta-cells in neonate and infant. Hirschsprung’s disease (HD) is also a rare disease in which infants show severe bowel movement disorder. We herein report an extremely rare case of combined CH and HD.

**Case presentation:**

The patient was a full-term male infant who showed poor feeding, vomiting, and hypotonia with lethargy on the day of birth. He was transferred to tertiary hospital after a laboratory analysis revealed hyperinsulinemic hypoglycemia. The patient showed remarkable abdominal distension without meconium defecation. An abdominal X-ray showed marked dilatation of the large bowel. He was diagnosed with CH (nesidioblastosis) associated with suspected HD. He was initially treated with an intravenous infusion of high-dose glucose with the intermittent injection of glucagon. This was successfully followed by treatment with diazoxide and octreotide (a somatostatin analog). At 8 months of age, HD was confirmed by the acetylcholinesterase staining of a rectal mucosal biopsy specimen, and a transanal pull-through operation was performed to treat HD. At 14 months of age, subtotal pancreatectomy was performed for the treatment of focal CH located in the pancreatic body. His postoperative course over the past 12 years has been uneventful without any neurologic or bowel movement disorders.

**Conclusions:**

Although it is extremely rare for CH to be associated with HD, associated HD should be considered when a patient with CH presents severe constipation.

## Background

Congenital hyperinsulinism (CH) is a rare disease characterized by persistent hyperinsulinemic hypoglycemia in neonates and infants. Patients with CH who receive inadequate treatment are at risk of developing permanent brain damage [1]. On the other hand, infants with Hirschsprung’s disease (HD) shows severe bowel movement disorder, and fatal enterocolitis may occur if patients are not appropriately treated [2]. An association between CH and HD has not been reported in the English literature. We present the first report of an extremely rare case in which a patient with both CH and HD was successfully treated.

## Case presentation

**Fig. 1:**
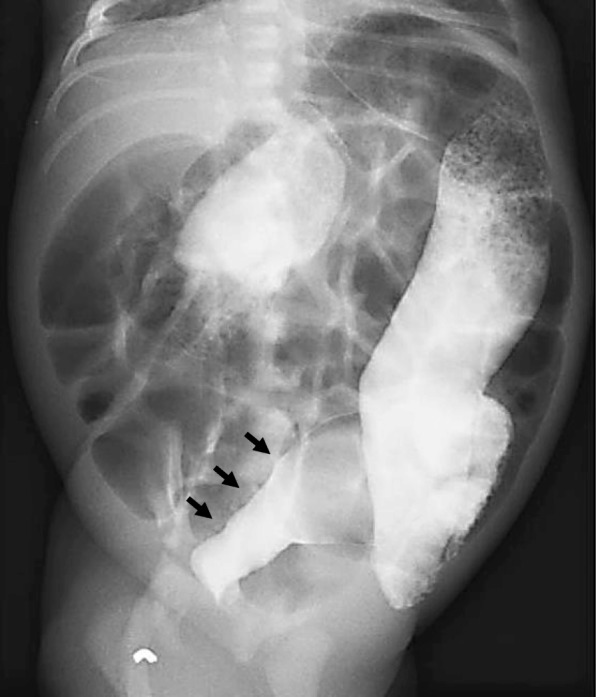
Contrast enemas showed a narrowed segment and caliber (arrow) in the rectosigmoid colon

**Fig. 2:**
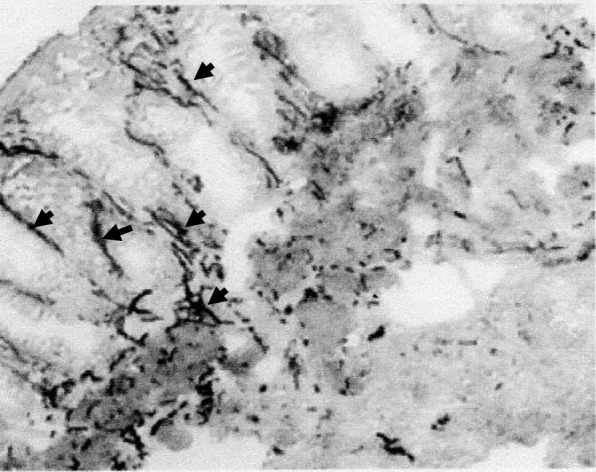
Immunohistochemical staining of acetylcholinesterase (AchE) in the rectal mucosa showed increased AchE staining of nerve fibers in the mucosal lamina propria and muscularis propria (arrow), and no ganglion cells were observed in the submucosal Meissner’s plexus

**Fig. 3:**
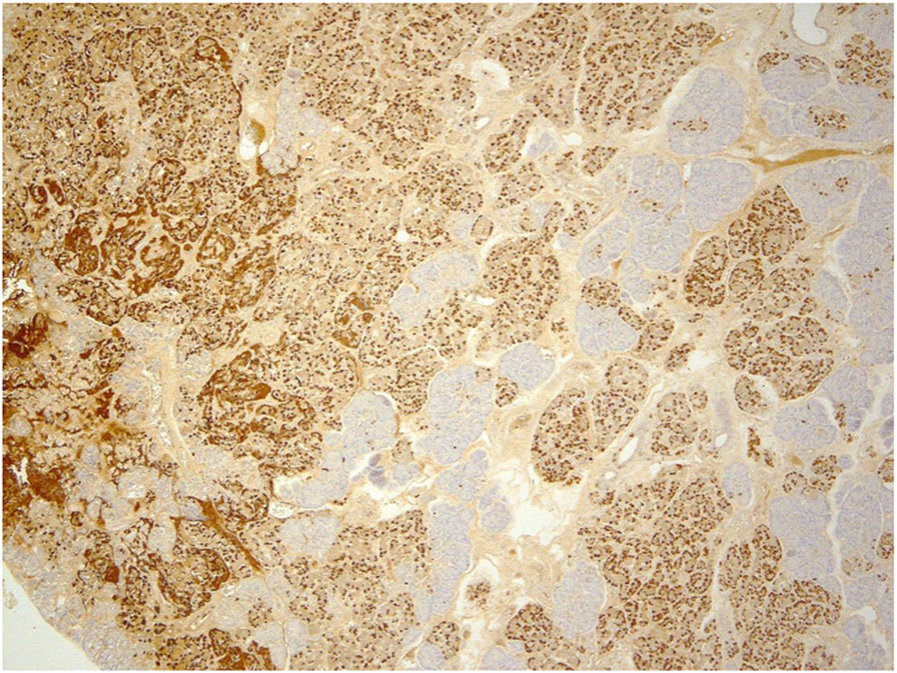
Immunohistochemical staining of insulin showed increased number of insulin-positive cells in the focal lesion of the resected pancreas specimen (dyed dark)

A male infant weighing 3870 g was born at 40 weeks’ gestation after an uneventful pregnancy. He showed poor feeding, vomiting, and hypotonia with lethargy on the day of birth. He was transferred to our hospital after a blood examination showed severe hypoglycemia. The patient’s blood levels of insulin and glucose were 15–20 μU/ml and 25–30 mg/dl, respectively. He also showed remarkable abdominal distension, and defecation of meconium did not occur until the second day after birth. An abdominal X-ray showed intestinal dilatation without gas in the pelvic cavity. He was first diagnosed with CH associated with suspected HD. He had no family history of CH or HD. The hypoglycemia was initially treated with an intravenous infusion of high-dose glucose (10 mg/kg/min) and the intermittent injection of glucagon (1 mg); then, the diazoxide (8 mg/kg/day) was initiated and the dose of diazoxide was gradually increased to 20 mg/kg/day. The patient was responsive to diazoxide and showed clinical improvement. The diagnosis of HD could not be confirmed in the neonatal period, because an increase of acetylcholinesterase (AchE)-positive nerve fibers was not noted in a biopsy specimen of the rectal mucosa. At 1 month of age, diazoxide was changed to the continuous injection of octreotide (0.5–1.2 μg/kg/h), because the adverse effects of diazoxide include fluid retention and congestive cardiac failure. Thereafter, octreotide was shown to be effective and the glucose infusion dosage could be gradually decreased and safely suspended; then, the patient was discharged from our hospital at 3 months of age. At home, the continuous subcutaneous injection of the octreotide was continued with the intermittent injection of glucagon to prevent hypoglycemia. The bowel movement disorder was treated by transanal colonic irrigation. A molecular examination revealed no mutations in the SUR1/Kir6.2 genes. At 8 months of age, short-type HD was confirmed by repeated biopsy of the rectal mucosa, which showed increased the AchE-positive nerve fibers in the mucosal lamina propria and muscularis propria of the rectosigmoid colon (Fig. 1), and contrast enema showed a narrow segment in the rectum (Fig. 2). A transanal pull-through operation was then successfully performed to treat the patient’s HD. After the operation, a pathological examination revealed a 7-cm aganglionic segment in the resected bowel. At 14 months of age, his family indicated that they hoped for surgical management to treat the patient’s CH, because it became difficult to continuously administer subcutaneous injections of octreotide at home. Although the focal lesion of CH could not be detected before surgery on MRI or US, the pancreatic body was outlined in both intraoperative ultrasound and histological examinations. Tissue specimens were obtained from the several points of the pancreas where the irregular arrangements of the pancreatic ducts were suspected through the intraoperative ultrasound. Then, we could outline the site of resection in the pancreas, and subtotal pancreatectomy was performed at the level of the superior mesenteric vein (SMV). After the operation, a pathological examination revealed that the focal lesion of CH had been completely resected (Fig. 3). Postoperatively, octreotide treatment was suspended without any hypo- or hyperglycemic events. In the 12 years after surgery, the patient’s course has been uneventful without any neurologic or bowel disorders.

## Discussion

CH has been described by various terms, including nesidioblastosis, persistent hyperinsulinemic hypoglycemia of infancy (PHHI), and persistent hyperinsulinemic and hypoglycemia (PHH). Inadequate treatment of hypoglycemia causes serious brain damage in patients with CH [1, 3, 4]. The estimated incidence of sporadic CH is 1 in 50,000 live births [3, 4]. CH has rarely been reported to be associated with clinical syndromes, including—but not limited to—Beckwith-Wiedemann, Kabuki, Sotos, and congenital central hypoventilation (Ondine) syndrome [4, 5]. Histopathological studies have revealed that there are foal and diffuse types of CH [1, 3–5]. Focal-type CH shows focal adenomatous hyperplasia of islet beta-cells associated with enlarged cytoplasm with abnormally large nuclei, and sometimes mutations of the SUR1 and Kir6.2 genes [3, 5]. In diffuse-type CH, hyperplasia of islet beta-cells is observed throughout the pancreas [1, 4–6]. These conditions have been rarely reported in adult patients after gastric surgeries [7, 8]. The causes of adult nesidioblastosis remain unknown, although several genetic defects have been reported in CH [3, 4, 9]. In particular, mutations of the SUR1 and Kir6.2 genes are frequently reported as causes of CH and are detected in 40–45% of all cases [4], although the disease is unrelated to either of these mutations in approximately half of CH patient [3, 9]. In the management of CH, conservative treatment is initially applied with high-dose glucose infusion, glucagon injection, diazoxide, and octreotide [1, 3, 4]. Surgical treatment is used to treat both the diffuse and focal types of CH when medical and dietary therapies are ineffective [1, 3–6]. In diffuse-type of CH, ≥ 95% subtotal pancreatectomy has been recommended for treating hypoglycemia; however, a high incidence of postoperative diabetes mellitus has been reported in long-term follow-up [4, 6, 7]. Adzick et al. suggested that the incidence of diabetes mellitus increased with long-term follow-up and that 47% of 189 patients who underwent near-total (> 95%) pancreatectomy had diabetes at 10–20 years of age [6]. On the other hand, partial resection of the pancreas is performed for most patients with focal type of CH and postoperative diabetes mellitus has rarely been reported. Recently, focal lesions have been frequently detected by 18-fluoro-dihydroxy phenylalanine (^18^F-DOPA) PET [10].

HD is another rare congenital disorder of bowel movement that is characterized by severe constipation and enterocolitis. The incidence of sporadic HD is 1 in 5000 live births [2, 11]. The absence of enteric ganglion cells at both the myenteric and submucosal plexuses, from the rectum to the proximal bowels, causes severe movement disorders in the affected bowel [2, 11]. Preoperatively, HD is definitively diagnosed by immunohistochemical staining of AchE-positive nerve fibers in the rectal mucosal biopsy specimens. In HD, increased AchE-positive fibers are observed in the mucosal lamina propria and muscularis propria and/or submucosa [11, 12]. However, increased AchE-positive fibers are not observed in all HD patients during the neonatal period; thus, AchE staining must be repeated later to obtain the definite diagnosis of HD [12]. Associated anomalies have been reported in HD, including Down syndrome, cardiac anomalies, mental retardation, anorectal malformation, and Ondine’s syndrome [2, 11]. However, no reports have described an association between CH and HD. Meissner et al. [13] reported one case in which CH was associated with congenital central hypoventilation syndrome (Ondine’s syndrome) and suggested that patients with Ondine’s syndrome might often manifest some clinical symptoms of autonomic nervous dysfunction, including HD and/or severe constipation [13]. Although the present case was not associated with Ondine’s syndrome, CH and HD may have common etiologies that are currently unknown. In addition to dysfunction of the enteric nervous system, as seen in HD, more complex molecular mechanisms may be involved in CH. HD is suggested to be caused by defective neural crest cell development, termed “neurocristopathy,” which causes dysfunction of the autonomic nervous system [14]. The PHOX2B polyalanine repeat mutation has also been identified in HD associated with Ondine’s syndrome [14]. Pancreatic islets cells can also be affected by the autonomic nervous system, as they are richly innervated by parasympathetic, sympathetic, and sensory nerves. Hennewig et al. reported a heterozygote missense mutation (Gly68Cys) in the PHOX2B gene in a rare case of Ondine’s syndrome associated with CH [15]. We speculated that the dysfunction of the autonomic nervous system derived from neurocristopathy may induce CH associated with HD, and an unknown de novo mutation in the PHOX2B gene may be involved in neurocristopathy which causes both HD and CH, without symptoms of CCHS.

In the present case, the focal lesion of CH could not be detected by MRI and/or US preoperatively, and ^18^F-DOPA PET could not be performed in our hospital at that time. According to the intraoperative examinations, the focal lesion could be outlined in the pancreatic body and subtotal pancreatectomy was performed at the level of the SMV. The pancreatic anatomy varies, and pancreatectomy at the level of the SMV has been suggested to be 53.5–75% pancreatectomy [1, 16]. If a 53.5–75% pancreatectomy had been ineffective, we would have performed additional pancreatic resection. Fortunately, however, the pathological examination revealed that the focal lesions of CH were all included within the resected specimen, and the patient showed normoglycemia without octreotide after surgery. Although postoperative diabetes mellitus and/or bowel movement disorders were not observed for 12 years after the operation, further follow-up is necessary to detect postoperative complications at an early stage.

## Conclusions

We report a successfully treated rare case of CH associated with HD. If a patient with CH presents severe constipation, the possibility of HD should be considered.

## Data Availability

All datasets supporting the conclusions of this article are included in the article.

## Abbreviations

Kir6.2: Potassium pore 6.2
MRI: Magnetic resonance imaging
PET: Positron emission tomography
SUR1: Sulfonylurea receptor 1
